# CONDUCTING COMMUNITY-BASED GERONTOLOGY PROJECTS IN UNDERGRADUATE CLASSROOMS: CAPSTONE CASE STUDIES

**DOI:** 10.1093/geroni/igad104.1944

**Published:** 2023-12-21

**Authors:** Ronald Berkowsky

**Affiliations:** California State University Channel Islands, Camarillo, California, United States

## Abstract

Collaborations between university-level students and community-based organizations have long shown to be mutually beneficial in that they can address pressing community needs, advance the mission of the organization, and enhance the learning of students. In this presentation, I describe project collaborations between a non-profit volunteer caregiving organization based in Ventura County (“Caregivers”) and Health Science undergraduate students (N = 76) enrolled in Capstone courses at California State University Channel Islands between 2022-2023. Projects were designed utilizing a community-based approach wherein the community partner (“Caregivers”) played an active and central role in determining project goals and outcomes. Four projects, each addressing and/or serving a specific need, were completed by different Capstone sections: (1) the creation of an online governance training program to educate high school and university students on the basics of leadership in nonprofits which serve older adults; (2) the creation of a series of videos to educate on and advocate for volunteer caregiving; (3) the analysis of quantitative and qualitative survey data to evaluate the experiences and satisfaction levels of local volunteer caregivers and care recipients; and (4) the creation of a toolkit to be used by volunteer caregiving organizations implementing community meal delivery programs for older adults. In this presentation, I detail each project along with their major successes and challenges as assessed through personal observation, feedback provided by the community partner, and student reflections. I conclude with discussing best practices in implementing community-based projects in undergraduate settings and describe the value of such projects across all stakeholders.

